# The Hidden Layer of MicroRNA Regulation in Gynecologic Cancers: IsomiRs, Arm Switching, and RNA Epitranscriptomic Modifications

**DOI:** 10.3390/ijms27167363

**Published:** 2026-08-18

**Authors:** Yussel Pérez-Navarro, César López-Camarillo, Laura C. Flores-García, María Elizbeth Alvarez-Sánchez, Alfredo Campoy Ramírez, Yarely M. Salinas-Vera

**Affiliations:** Posgrado en Ciencias Genómicas, Universidad Autónoma de la Ciudad de México, Ciudad de México 03104, Mexico; yussel.perez@estudiante.uacm.edu.mx (Y.P.-N.); cesar.lopez@uacm.edu.mx (C.L.-C.); lcfloresg@hotmail.com (L.C.F.-G.); maria.alvarez@uacm.edu.mx (M.E.A.-S.); alfredo.campoy@alumnos.uacm.edu.mx (A.C.R.)

**Keywords:** miRNAs, gynecologic cancers, epitranscriptomics, isomiRs, arm switching

## Abstract

MicroRNAs (miRNAs) are key regulators of gene expression that act primarily by binding to target messenger RNAs (mRNAs). However, the biology of miRNAs is more complex than initially thought, with functional complexity extending beyond canonical sequences. A multilayered miRNA regulatory landscape involving isomiR generation, altered 5p/3p strand usage, arm switching, A-to-I RNA editing, and epitranscriptomic RNA modifications operates in eukaryotic cells to regulate miRNA function. Collectively, these mechanisms expand the functional diversity of miRNAs by regulating their biogenesis, stability, strand selection, and target specificity, increasing their functional plasticity and contributing to regulatory heterogeneity found in cells. IsomiRs arise from alternative Drosha/Dicer processing, terminal nucleotide additions, RNA editing, and genetic variation, producing functionally distinct isoforms. Arm switching alters gene regulatory outputs through context-dependent changes in predominant 5p/3p strand usage. In addition, epitranscriptomic RNA modifications, such as m6A and m5C, together with A-to-I RNA editing, represent an additional layer of miRNA regulation. These mechanisms can act directly on miRNAs or their precursors, or indirectly by modifying circRNAs and lncRNAs, thereby altering miRNA availability and function. Together, these processes form a dynamic regulatory network that influences key cancer hallmarks, including cell proliferation, apoptosis, epithelial–mesenchymal transition, metastasis, immune evasion, and therapy resistance. However, the contribution of these non-canonical regulatory layers to tumor-specific miRNA function remains poorly understood. In this review, we explore how isomiR generation, miRNA strand selection, arm switching, and epitranscriptomic regulation expand the functional diversity of miRNAs in gynecologic cancers.

## 1. Introduction

MicroRNAs (miRNAs) are small non-coding RNAs that regulate gene expression at the post-transcriptional level by modulating the translation and stability of target messenger RNAs (mRNAs) [1,2]. These regulatory functions are fundamental for cellular processes such as cell proliferation, differentiation, apoptosis, and migration. Dysregulation of miRNA expression is associated with tumor progression in various types of human malignancies, including gynecologic cancers such as ovarian, cervical, and endometrial cancer [3]. Likewise, miRNAs have emerged as diagnostic and prognostic biomarkers and as potential therapeutic targets in molecular oncology [4]. However, the biology of miRNAs is more complex than initially thought. This complexity is particularly relevant in gynecologic cancers, where molecular heterogeneity, histological diversity, and differences in therapeutic response complicate the interpretation of canonical miRNA expression profiles. Recent studies have described isomiRs, variants of miRNAs generated by differences in cleavage at the 5′ or 3′ ends, nucleotide additions, or RNA editing, which can alter target selection and the functional activity of miRNAs. Importantly, isomiRs are not merely sequencing artifacts but functionally relevant molecules with distinct regulatory properties [5]. On the other hand, the arm-switching phenomenon, in which predominant 5p/3p strand usage changes between biological contexts, increases the functional plasticity of miRNAs and contributes to regulatory heterogeneity in tumor cells [6].

Additionally, epitranscriptomic RNA modifications, including N6-methyladenosine (m6A), 5-methylcytosine (m5C), and Adenosine-to-Inosine (A-to-I) RNA editing, constitute another regulatory layer that affects miRNA maturation, stability, loading into the RNA-induced silencing complex (RISC), and target specificity [7,8]. Experimental studies, primarily from cancer models, suggest that these modifications influence miRNA maturation and activity, although their specific contributions in gynecologic cancers remain incompletely characterized [7,9]. Therefore, understanding this hidden layer of miRNA regulation is essential for accurately interpreting miRNA function in gynecologic tumors. Although gynecologic malignancies are heterogeneous, this review primarily focuses on ovarian, cervical, and endometrial cancers, as these tumor types are the primary contexts in which the non-canonical miRNA regulatory mechanisms discussed herein have been investigated. In this review, we discuss the molecular mechanisms that contribute to miRNA diversity, including isomiR generation, differential 5p/3p arm usage, arm switching, and epitranscriptomic regulation. We further examine the functional implications of these regulatory mechanisms in gynecologic cancers and discuss their emerging clinical relevance, as well as the biological and methodological challenges that remain unresolved.

## 2. Literature Search Strategy

A literature search was conducted in PubMed/MEDLINE to identify relevant studies on the biology, regulation, and function of miRNAs in gynecologic cancers, with particular emphasis on mechanisms of miRNA biogenesis, isomiRs, arm switching, and epitranscriptomic modifications. The search covered publications available from database inception to 15 May 2026, when the final literature search was performed. The main search terms included combinations of the following keywords: “microRNA”, “miRNA”, “miRNA biogenesis”, “miRNA maturation”, “miRNA processing”, “isomiRs”, “isomiR profiling”, “miRNA isoforms”, “non-canonical miRNAs”, “arm switching”, “miRNA arm switching”, “miRNA strand switching”, “5p/3p switching”, “miRNA strand selection”, “epitranscriptomics”, “RNA modifications”, “m6A”, “m5C”, “m1A”, “A-to-I editing”, “RNA editing”, “RNA methylation”, “METTL3”, “ALKBH5”, and “gynecologic cancer”, including ovarian, cervical, and endometrial cancers. Relevant research articles and review articles were considered based on their contribution to understanding miRNA regulation and associated mechanisms in gynecologic cancers.

## 3. Overview of miRNA Biology in Cancer

MiRNAs are critical post-transcriptional regulators that modulate gene expression by repressing translation or degrading mRNA [1,10]. The function of miRNAs depends not only on their quantity but also on the variety generated by canonical and non-canonical biogenesis pathways, sequence heterogeneity, and differential selection of the 5p or 3p arm [5,6]. Therefore, alterations occurring during miRNA maturation represent potential sources of functional diversity that may influence tumor biology beyond changes in miRNA abundance.

### 3.1. Canonical miRNA Biogenesis

**Figure 1:**
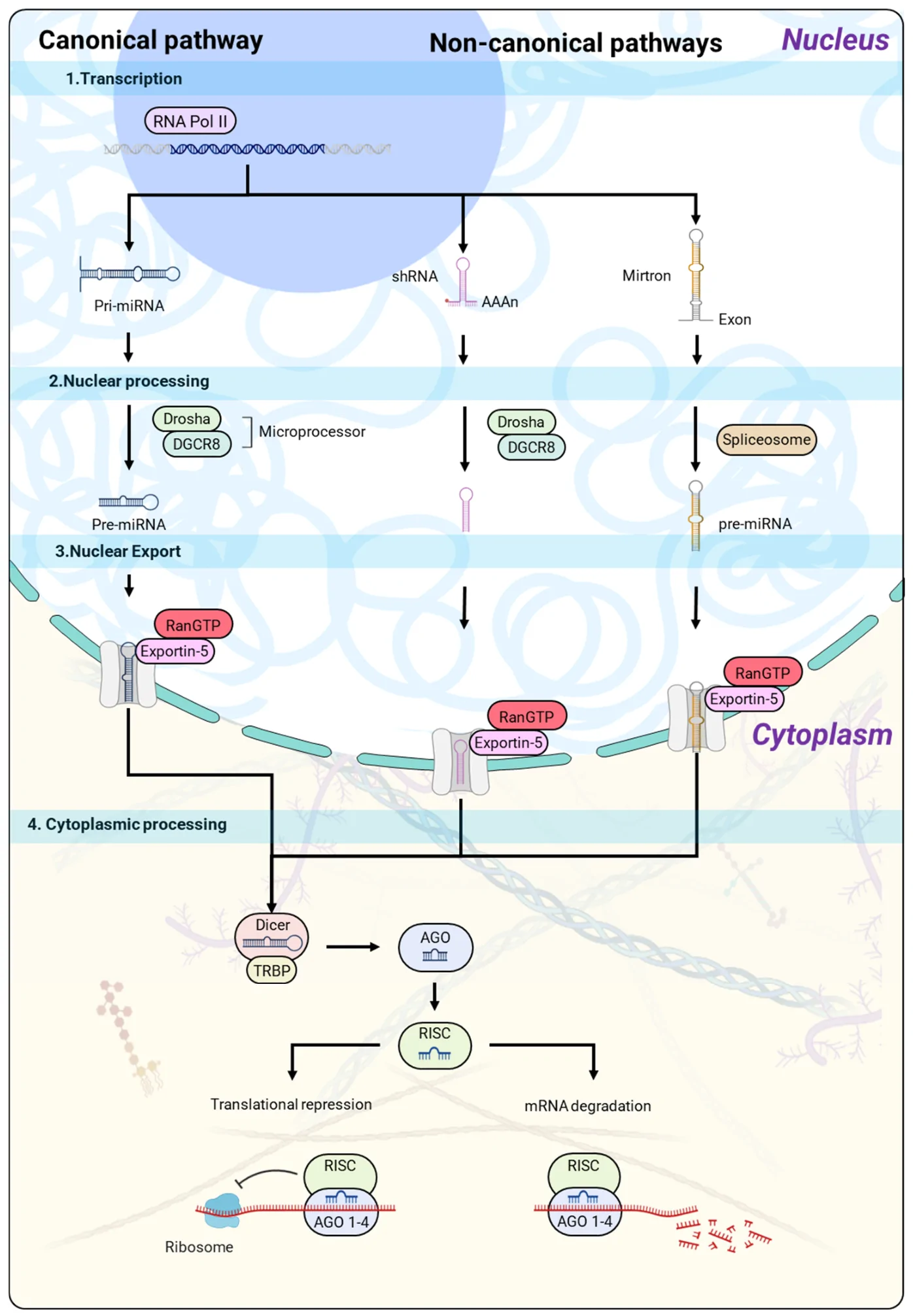
Canonical and non-canonical pathways of miRNA biogenesis. In the canonical pathway, miRNAs are transcribed by RNA polymerase II as pri-miRNAs and processed in the nucleus by the Drosha/DGCR8 microprocessor complex to generate pre-miRNAs. These are exported to the cytoplasm via Exportin-5/Ran-GTP and subsequently cleaved by Dicer to produce mature miRNA duplexes. One strand is loaded into the AGO-containing RISC, while the passenger strand is degraded. In non-canonical pathways, miRNAs originate from mirtrons or shRNA-derived transcripts that bypass Drosha processing and converge at downstream cytoplasmic steps, where they are processed by Dicer or directly incorporated into AGO complexes. In both cases, functional RISC complexes are formed, mediating post-transcriptional gene regulation through mRNA degradation or translational repression.

The canonical biogenesis of miRNAs is a highly regulated and conserved process in eukaryotes that ensures the production of mature and functional miRNAs capable of regulating gene expression [2,11] (Figure 1). First, the genes that encode miRNAs are transcribed by RNA polymerase II as primary miRNAs (pri-miRNAs), which are variable-length molecules containing stem-loop structures essential for their recognition and processing [12,13]. These hairpins contain sequences that will eventually form the seed region of the mature miRNA, which is responsible for its specificity toward target genes. Once synthesized, the pri-miRNAs are processed in the nucleus by the microprocessor complex, composed of the RNase III enzyme Drosha and its cofactor, DiGeorge syndrome critical region 8 (DGCR8). Drosha makes a precise cut at the ends of the hairpin, generating a precursor miRNA (pre-miRNA) of approximately 70 nucleotides, with 2-nucleotide overhangs at the 3′ end [14]. This step is critical; any alteration in the expression or function of Drosha or DGCR8 can affect the ratio of tumor-suppressor and oncogenic miRNAs, with direct implications for tumor progression [15,16]. Subsequently, pre-miRNAs are transported to the cytoplasm via Exportin-5 in a Ran-GTP-dependent process, thereby protecting them from degradation and making them accessible for final maturation [17]. In the cytoplasm, the ribonuclease III Dicer recognizes the ends of the pre-miRNA and positions its catalytic domains to make a precise cut near the terminal loop, releasing a mature miRNA duplex of approximately 22 nucleotides with two-nucleotide overhangs at the 3′ end [18]. From this dimer, one strand is incorporated into the RISC, while the complementary strand is degraded. The selected strand, known as the guide strand, determines the specificity of the miRNA toward the target mRNAs and its ability to regulate gene expression [19]. Although Dicer can process pre-miRNAs independently, its efficiency and accuracy are modulated by cofactors such as TAR RNA-binding protein (TRBP). TRBP binds to the RNA duplex, stabilizes its interaction with Dicer, and improves the fidelity of the cut, thereby influencing the selection of the guide strand and the generation of functional variants known as isomiRs [20]. Additionally, TRBP connects Dicer to Argonaute proteins (AGO1-4), facilitating RISC assembly and guiding the active strand to its target mRNAs [21].

In gynecologic cancers, the biogenesis machinery of miRNAs may be dysregulated, directly affecting mature miRNA expression and the regulation of critical genes. Several studies have documented that the expression of Drosha, Dicer, and DGCR8 is altered in ovarian, endometrial, and cervical cancers, which correlates with aggressive tumor characteristics and a poor prognosis. For example, in epithelial ovarian cancer (EOC), reduced expression of Dicer and Drosha is associated with advanced stages, suboptimal response to surgery, and lower overall survival; high expression of both genes is correlated with greater survival [22,23]. Likewise, in endometrial cancer, studies of clinical samples showed that Dicer and Drosha expression is significantly lower than in normal tissue, and their decrease is associated with a high histological grade and certain clinical factors, such as parity and body mass index (BMI) [24]. Additionally, in cervical cancer, Drosha and Dicer are overexpressed in human papillomavirus type 16 (HPV16) positive tumors, and their regulation may be mediated by the viral oncoproteins E6/E7, which alter the overall expression of miRNAs [25]. Furthermore, studies examining copy-number gains on the short arm of 5p have highlighted Drosha as one of the overexpressed genes, indicating that genomic changes may disrupt the miRNA machinery [26].

These combined findings indicate that any disruption of the canonical biogenesis pathway can alter the profiles of tumor-suppressor and oncogenic miRNAs, thereby affecting proliferation, invasion, and therapy resistance. Therefore, Drosha, Dicer, and DGCR8 represent potential prognostic biomarkers and therapeutic targets in gynecologic cancers [27,28]. Although canonical miRNA biogenesis generates highly conserved mature miRNAs, current understanding suggests that additional regulatory layers, including alternative processing, nucleotide modifications, and sequence variation, can influence the functional diversity of miRNAs in cancer.

### 3.2. Non-Canonical miRNA Biogenesis

In addition to the canonical pathway, miRNAs can also be generated through alternative non-canonical pathways that use different processing mechanisms. Unlike the canonical pathway, these routes do not necessarily require all components of the canonical machinery and may bypass or retain specific steps depending on the pathway involved, including Drosha, Dicer, Exportin-5, or AGO2 [29]. These pathways constitute an adaptive mechanism that broadens the functional diversity of miRNAs and enhances tumor cells’ ability to regulate gene networks, even when canonical biogenesis is compromised (Figure 1) [29]. Among these pathways, mirtrons represent a paradigmatic example. They originate from spliced introns that adopt recognizable hairpin structures that are recognized by Dicer, thereby bypassing Drosha processing [30,31]. Mirtrons have been estimated to account for approximately 15% of the human miRNA repertoire and exhibit specific structural features, such as prominent ends and variability in Dicer cleavage sites, which enable the generation of isomiRs and broaden the functional heterogeneity of miRNAs [29]. Their high single nucleotide polymorphism (SNP) density and patterns of positive selection indicate that mirtrons constitute an intrinsic source of regulatory variability.

On the other hand, other non-canonical precursors include miRNAs derived from tRNAs, snoRNAs, and endogenous short hairpin RNAs, which produce functional fragments capable of being incorporated into RISC or directly modulating translation at the ribosomal level [32,33]. The cleavage position depends on the three-dimensional conformation of the RNA molecule, which determines the functional strand and the stability of the mature miRNA. A special subset of pre-miRNAs is the simtrons, which are produced independently of classical splicing and the usual biogenesis components, such as DGCR8, Exportin-5, or AGO2, but require Drosha for their efficient maturation in vitro [31]. The simtrons are incorporated into RISC and can exert functional silencing, which represents a hybrid pathway that combines independence from the canonical route with partial dependence on Drosha [34]. Additionally, alternative miRISC complexes dependent on Argonaute RISC Catalytic Component 3 (AGO3) and DExH-Box Helicase 9 (DHX9) have been described that are capable of recognizing mRNAs harboring coding-sequence (CDS) mutations. This mechanism, demonstrated in ovarian granulosa cell tumors, directs the selective degradation of mutant mRNAs, expanding the functionality of miRNAs beyond the classic RISC and demonstrating their capacity to mediate highly specific gene regulation [35]. In contrast, in cervical cancer, evidence of non-canonical biogenesis focuses on alterations in Drosha and Dicer mediated by HPV16 oncoproteins or by copy-number gains on 5p, which affect the overall expression of miRNAs [25].

Finally, non-canonical biogenesis contributes to the generation of isomiRs and arm-switching phenomena, since variants derived from mirtrons, tRNAs, snoRNAs, or simtrons can be processed differently, yielding strands with variable ends and distinct functional preferences. This functional heterogeneity confers regulatory plasticity and is particularly relevant in gynecologic tumors, where the canonical pathway may be partially or completely compromised [7].

### 3.3. From Canonical miRNAs to miRNA Heterogeneity

Although canonical biogenesis provides an accurate framework for the production of mature miRNAs, the functional reality of these molecules is considerably more complex. The heterogeneity of miRNAs arises from multiple sources, including variations in cleavage sites by Drosha and Dicer, post-transcriptional modifications, and non-canonical pathways. This diversity leads to isomiRs, variants of the same miRNA that differ by one or more nucleotides at the 5′ or 3′ ends, and to altered 5p/3p strand usage, including arm switching, a process characterized by context-dependent changes in predominant strand usage [36,37].

The generation of isomiRs and the selection of the guide strand depend on precisely regulated molecular factors, such as the thermodynamic stability of the ends, the secondary structure of the pre-miRNA hairpin, interactions with cofactor proteins like TRBP, and competition for incorporation into RISC, which determines which variants are stabilized and perform regulatory functions [20,21]. This process is modulated by epitranscriptomic modifications, such as m6A, m5C, and A-to-I editing, which affect both the maturation and stability of miRNAs, further increasing functional heterogeneity [7,38]. Therefore, functional heterogeneity reflects not only differences in sequence but also in the selection of gene targets and in silencing efficiency. For example, variants of the same miRNA can have different affinities for specific mRNAs, modulating different cellular pathways [39]. In gynecologic cancers, this diversity allows tumor cells to adapt to microenvironmental and therapeutic stimuli, which contributes to progression, invasion, and drug resistance [6,40]. Consequently, the transition from a model of canonical miRNAs to an understanding of functional heterogeneity is essential for correctly interpreting tumor expression profiles, designing miRNA-based diagnostic strategies, and developing therapies that account for the diversity of functional variants within the same cell type.

## 4. Molecular Diversity and Regulation of IsomiRs 

Gynecologic cancers, including ovarian, cervical, and endometrial malignancies, are a leading cause of cancer-related morbidity and mortality in women worldwide. Current knowledge indicates that post-transcriptional regulatory mechanisms, particularly the generation of miRNA isoforms known as isomiRs, contribute to the molecular heterogeneity observed in these tumors [41].

### 4.1. Definition and Classification of isomiRs

The discovery of miRNAs revolutionized our understanding of post-transcriptional gene regulation. High-throughput sequencing technologies have revealed that mature miRNAs are not represented by a single invariant sequence. Instead, each miRNA locus gives rise to a heterogeneous population of closely related isoforms, collectively known as isomiRs, which differ from the canonical miRNA sequence in length, nucleotide composition, or both [42,43]. The term isomiR was originally coined to describe sequence variants derived from the same miRNA precursor that exhibit modifications at the 5′ or 3′ ends, internal nucleotide substitutions, or post-transcriptional nucleotide additions [44]. Subsequent studies have demonstrated that isomiRs are not merely sequencing artifacts or byproducts of imprecise processing, but biologically regulated molecules that are loaded into Argonaute-containing RISCs, interact with target mRNAs, and actively participate in gene regulatory networks [43,45]. IsomiR generation is intrinsically linked to miRNA biogenesis. Canonical miRNA maturation involves sequential cleavage of pri-miRNAs by the Drosha-DGCR8 microprocessor complex in the nucleus and by Dicer in the cytoplasm, yielding mature miRNA duplexes from either the 5p or 3p arm of the precursor hairpin [46,47]. Variations in Drosha or Dicer cleavage sites, exonuclease-mediated trimming, terminal nucleotide transferase activity, RNA editing, and genetic polymorphisms can produce multiple mature isoforms from a single precursor molecule [48,49]. Consequently, a single pre-miRNA may generate multiple distinct isomiRs, and in some cases, the most abundant isoform differs from the canonical sequence annotated in miRBase [50,51]. Large-scale sequencing across over 10,000 tumor and normal samples from TCGA revealed 7466 isomiRs arising from 807 miRNA arms and 767 loci, demonstrating that isomiR production is a ubiquitous feature of human miRNA biology rather than an exceptional phenomenon [51]. Notably, only a small fraction of these variants are shared across tissues and disease states, highlighting the tissue specificity and context dependency of isomiR expression, which is further influenced by developmental stage, disease subtype, sex, ethnicity, and cellular environment [51,52].

Several classification systems have been proposed, with the most widely used classification dividing isomiRs into 5′ isomiRs, 3′ isomiRs, polymorphic isomiRs, and mixed isomiRs [44,53,54]. 5′ isomiRs contain additions or deletions at the 5′ end, altering the miRNA seed sequence and redirecting the miRNA toward distinct target mRNAs. Functional studies demonstrate that highly expressed 5′ isomiRs can regulate distinct biological pathways and exert effects that differ from those of canonical miRNAs; for example, 5′ isomiRs of miR-140-3p display tumor-suppressive activities by targeting genes distinct from those of canonical miR-140-3p [44]. 3′ isomiRs are the most prevalent and typically preserve the canonical seed sequence, yet modifications at the 3′ end can influence miRNA stability, RISC loading, intracellular localization, and target-binding affinity [42,50]. Genome-wide analyses indicate that 3′ heterogeneity is more common than 5′ heterogeneity, with approximately 40–50% of detected variants corresponding to 3′ modifications [42,55]. A particularly important subgroup includes variants with non-templated nucleotide additions (NTAs), such as mono-adenylation or mono-uridylation, which account for roughly 80% of terminal additions [56,57]. Adenylation generally increases miRNA stability, whereas uridylation often promotes degradation and can influence arm selection and target specificity [50,57].

Polymorphic isomiRs contain internal nucleotide substitutions that do not alter length, arising from SNPs, RNA editing, or other post-transcriptional modifications. The most studied form is A-to-I editing, catalyzed by adenosine deaminase acting on RNA (ADAR) enzymes, which can substantially alter miRNA function, especially within the seed region [43]. Mixed isomiRs exhibit both terminal length variations and internal substitutions, combining features of terminal and polymorphic variants and potentially targeting unique gene sets [42,58].

Overall, isomiRs are not passive byproducts of miRNA processing; rather, they are integral components of the miRNA regulatory network. Their diversity enables modulation of target recognition, stability, arm selection, and intracellular function, influencing cell differentiation, apoptosis, immune responses, and cancer progression. Recognition of the functional significance of isomiRs has important implications for their use as biomarkers, therapeutic targets, and determinants of disease heterogeneity in gynecologic and other cancers (Table 1).

### 4.2. Mechanisms of isomiR Generation

The generation of isomiRs is a highly dynamic process that occurs throughout multiple stages of miRNA biogenesis and maturation, encompassing both canonical processing events and post-transcriptional modifications, which together expand the functional diversity of the miRNA transcriptome [43,44,56,59]. Variability in Drosha and Dicer cleavage constitutes the primary source of isomiR diversity, as subtle shifts in cleavage sites, influenced by sequence motifs, secondary structures, stem flexibility, and structural bulges, produce isoforms with shifted 5′ or 3′ termini that can be amplified during cytoplasmic maturation. These variations enable the generation of multiple functional isoforms from a single miRNA precursor, thereby significantly diversifying target recognition and regulatory capacity [57,60].

In addition to alternative cleavage, miRNAs undergo exonucleolytic trimming and remodeling at their 3′ ends, mediated by enzymes such as poly(A)-specific ribonuclease (PARN), which remove genomically encoded or post-transcriptionally added nucleotides. Terminal modifications further diversify miRNA sequences through NTAs, such as adenylation and uridylation, catalyzed by terminal nucleotidyl transferases including poly(A) RNA polymerase D4/germline development 2 (PAPD4/GLD2), poly(A) RNA polymerase D5 (PAPD5), mitochondrial poly(A) polymerase (MTPAP), terminal uridylyl transferase 1 (TUT1), terminal uridylyl transferase 4 (TUT4; also known as ZCCHC11), and terminal uridylyl transferase 7 (TUT7; also known as ZCCHC6). These modifications modulate miRNA stability, RISC loading, intracellular localization, and arm selection, and may alter strand preference and, in specific contexts, contribute to arm switching [56,57,61]. RNA editing, particularly A-to-I conversion mediated by ADAR1 and ADAR2, introduces internal nucleotide changes that can profoundly affect seed sequences, creating polymorphic isomiRs with alternative target repertoires. Although less frequent than terminal modifications, these editing events can produce substantial functional effects, effectively expanding the regulatory potential of a single miRNA locus. Differential strand selection further contributes to miRNA functional diversity. In specific contexts, changes in predominant strand usage may result in arm switching. This arm preference is modulated not only by terminal modifications such as TUT4/TUT7-mediated uridylation but also by interactions with RNA-binding proteins, including TAR DNA-binding protein 43 (TDP-43), TRBP, and protein activator of PKR (PACT), which influence the precision of Drosha and Dicer processing, thereby integrating multiple layers of post-transcriptional control [43,44,46,57]. Genetic variation within pre-miRNAs, including SNPs, adds another dimension of diversity by altering precursor secondary structure, cleavage site selection, and folding stability, thereby producing novel isomiRs that contribute to inter-individual variability, tumor heterogeneity, and differential cellular responses [62].

Together, alternative cleavage, exonucleolytic trimming, non-templated nucleotide additions, RNA editing, RNA-binding protein modulation, and genetic variation constitute a highly integrated network that governs isomiR generation. Far from being byproducts of imprecise processing, isomiRs represent a regulated and functional layer of post-transcriptional control, enhancing the complexity of miRNA-mediated gene regulation, enabling context-specific modulation of target genes, and contributing to cellular adaptation, tumor heterogeneity, and disease progression (Table 1).

### 4.3. IsomiRs in Gynecologic Cancers

IsomiR profiles are increasingly recognized as an additional layer of miRNA heterogeneity in gynecologic cancers, including ovarian, cervical, and endometrial tumors. These variants exhibit tumor-specific expression patterns that can modify miRNA target recognition and influence biological processes such as cell proliferation, apoptosis, migration, invasion, and survival.

Ovarian cancer, particularly EOC, is characterized by profound molecular heterogeneity that cannot be fully explained by the expression patterns of canonical miRNAs alone. Recent studies have highlighted that isomiRs constitute a substantial and functionally relevant component of the small RNA transcriptome in these tumors, contributing to tumor progression, metastasis, therapeutic response, and histological subtype specification. One of the most comprehensive analyses was conducted by Velle and coworkers, who profiled the complete miRNA/isomiR repertoire in 215 stage I EOC samples representing multiple histological subtypes. Among 971 detected miRNA transcripts, 349 (36%) corresponded to canonical reference miRNAs, while 622 (64%) were classified as isomiRs, predominantly arising from 3′-end modifications (iso_3p; 54%), followed by 5′-end modifications (iso_5p; 12%), non-templated nucleotide additions (iso_add; 6%), and mismatch variants (iso_snp; 2%). The iso_3p variants displayed expression levels comparable to or exceeding canonical miRNAs, indicating that they are abundant and functionally relevant. Moreover, 63% of differentially expressed miRNA transcripts across histological subtypes corresponded to isomiRs, including 42 histotype-specific miRNA biomarkers, many of which were isomiRs, characterizing aggressive subtypes such as high-grade serous ovarian carcinoma (HGSOC) and specific patterns in low-grade serous ovarian carcinoma (LGSOC). Among the overexpressed isoforms in HGSOC were miR-15b-3p,ref, miR-29a-5p,iso_3p:A, miR-454-3p,iso_3p:t, miR-19a-3p,iso_3p:a, miR-146b-5p,iso_3p:g, and miR-30e-5p,iso_3p:CT, whereas in LGSOC, miR-34c-5p,iso_3p:c and miR-34c-5p,iso_3p:gc predominated [63]. These findings demonstrate that isomiR profiling provides additional discriminatory information for molecular classification of ovarian tumors and may complement canonical miRNA-based analyses. The generation and regulation of isomiRs are strongly influenced by terminal uridyl transferases TUT4 and TUT7. In IGROV1 cells, catalytic inactivation of TUT4/7 drastically reduced uridylated isoforms, particularly mono-uridylated species, while selectively increasing adenylated isoforms within specific miRNA families, indicating highly regulated, context-dependent control of isomiR populations. This modulation affected cellular proliferation and migration, highlighting the functional impact of uridylation-dependent isomiRs in metastatic processes [57].

Additionally, RNA editing, primarily A-to-I conversion catalyzed by ADAR enzymes, constitutes another major source of isomiR diversity. In HGSOC, 13 recurrent editing sites were identified, nine of which occurred within seed regions, including miR-200b-3p, whose editing frequency was significantly higher in tumors than in normal ovarian tissue. Increased editing of miR-200b-3p correlated with poor patient survival and enhanced tumor aggressiveness, promoting proliferation, migration, and three-dimensional spheroid formation while redirecting target selection toward tumor suppressor genes such as MXI1 [64]. Large-scale analyses from TCGA and the Tumor IsomiR Encyclopedia (TIE) further support the relevance of isomiRs in ovarian cancer. Commonly detected variants include members of the miR-200 family, miR-21, miR-205, miR-182/183, miR-199, miR-30, miR-125b, miR-194, and miR-375, many of which are established regulators of epithelial-to-mesenchymal transition (EMT), invasion, metastasis, chemoresistance, and stemness. Differential expression analyses identified overexpressed isoforms such as miR-202-5p, miR-503-5p, miR-10a-5p, miR-10b-5p, miR-127-5p, and let-7 family members, and underexpressed variants including miR-200c-3p, miR-141-3p, miR-205-5p, miR-375, and miR-21 [42,65], illustrating extensive remodeling of the isomiR landscape during ovarian tumorigenesis.

Cervical cancer is one of the most extensively studied malignancies associated with aberrant microRNA regulation; the strong etiological link to persistent high-risk human papillomavirus (HPV) infection has highlighted the importance of miRNA dysregulation in tumorigenesis. Although canonical miRNAs have been widely characterized, recent high-throughput sequencing studies have revealed that isomiRs constitute an additional layer of post-transcriptional complexity, impacting tumor initiation, progression, metastasis, and therapeutic response [66,67]. The generation of isomiRs in cervical cancer occurs through multiple coordinated mechanisms. Alternative cleavage by Drosha and Dicer produces 5′- and 3′-isomiRs with modified seed sequences, thereby expanding the repertoire of target genes. Structural distortions within pri-miRNA stems can induce alternative Drosha cleavage, generating functional 5′-isomiRs with distinct seeds, such as miR-9-alt, which can regulate over 500 additional targets compared with canonical miR-9, demonstrating how alternative processing can substantially expand the regulatory networks of miRNAs in cervical tumor cells [66,68]. Furthermore, 3′ end remodeling contributes to isomiR diversity. PARN acts as both a trimmer and a de-tailer, regulating the maturation of multiple miRNAs, including miR-362-5p, miR-425-5p, miR-361-3p, miR-182-5p, miR-301a-3p, miR-224-5p, and miR-21. Loss of PARN results in accumulation of longer isoforms and increased adenylation, confirming that isomiR generation is a highly controlled regulatory mechanism rather than a byproduct of imprecise processing [49].

On the other hand, HPV-mediated disruption of p53 signaling further shapes the isomiR landscape. The miR-34 family, direct transcriptional targets of p53, is downregulated in HPV-positive cervical cancer, including miR-34a-5p, miR-34a-3p, miR-34b-5p, miR-34b-3p, miR-34c-5p, and miR-34c-3p, which inhibit proliferation, migration, and invasion. Notably, miR-34c-3p exhibits one of the strongest inhibitory effects, regulating invasion- and metastasis-associated genes such as Microtubule-associated protein 2 (MAP2), matrix metalloproteinase-2 (MMP2), and matrix metalloproteinase-9 (MMP9), underscoring the functional relevance of non-canonical strands and their associated isomiRs [47]. Large-scale analyses support the prevalence and functional significance of isomiRs in cervical cancer. According to the TIE, TCGA-CESC tumors display a characteristic isomiR landscape dominated by variants from the miR-21, miR-29, miR-183, miR-10a/b, miR-145, miR-148a, miR-151a, miR-26a, miR-126, miR-30, miR-143, miR-99b, miR-93, miR-182, miR-22, miR-205, miR-203, miR-375, miR-200, and let-7 families. Frequently detected isoforms include miR-183-5p|+1, miR-10a-5p|+1, miR-30e-5p|+1, miR-22-3p|+1, miR-192-5p|+1, miR-203a-3p|+1, and miR-142-3p|+1, indicating that 5′ and 3′ terminal modifications are widespread. Many of these miRNAs regulate EMT, angiogenesis, immune evasion, proliferation, and therapy resistance, suggesting that their isomiRs exert distinct regulatory functions during cervical tumor progression [42,65]. Differential expression studies have also identified cervical cancer-associated isomiRs, including upregulation of miR-320a-3p and miR-7704, and downregulation of miR-26a-5p, which may affect pathways that control cell cycle progression and apoptosis, thereby contributing to malignant transformation [69].

Taken together, evidence indicates that isomiRs are integral components of cervical cancer biology. Through alternative Drosha/Dicer processing, 3′ end trimming and tailing, adenylation, and HPV-mediated deregulation of tumor-suppressive miRNAs such as the miR-34 family, isomiRs expand the regulatory capacity of the miRNA network and influence key pathways controlling proliferation, apoptosis, migration, invasion, angiogenesis, and metastasis [49,65,70].

In endometrial cancer, recent studies have highlighted the significant contribution of isomiRs to post-transcriptional regulation. These variants add a layer of complexity beyond canonical miRNAs, influencing proliferation, invasion, differentiation, and pathways involved in hormone responsiveness and EMT, and contributing to tumor heterogeneity [42,65]. Evidence for the functional relevance of isomiRs in endometrial biology first emerged from studies on the miR-34/449 family, comprising six homologous miRNAs (miR-34a, miR-34b, miR-34c, miR-449a, miR-449b, and miR-449c) encoded across three distinct loci. High-throughput sequencing analyses have shown that these miRNAs generate numerous isomiRs, some more abundant than the corresponding canonical miRNAs, particularly the 5′-isomiRs derived from miR-34b and miR-449c, suggesting that alternative processing is a major source of functional diversity. These 5′-isomiRs possess altered seed regions, expanding the target repertoire of the miR-34/449 family and regulating pathways involved in endometrial differentiation and tissue remodeling [71]. Additionally, studies on endometrial receptivity during the transition from the proliferative to the mid-secretory phase identified 157 differentially expressed miRNAs and isomiRs, with members of the miR-34/449 family, including miR-449a-5p and several miR-449c-derived 5′-offset isoforms (miR-449c-5p_t_+1_0 and miR-449c-5p_t_+1_+1), showing the strongest regulation, while miR-449b-derived variants were absent. These findings underscore the role of specific isomiRs in regulating endometrial differentiation and tissue remodeling processes, which are often disrupted during malignant transformation [71]. Direct evidence of isomiR involvement in endometrial carcinogenesis was provided by Lu and coworkers, who compared isomiR profiles between normal human endometrial epithelial cells (HEC) and the endometrial adenocarcinoma cell line ISK. Their analysis revealed that each miRNA exists as a complex population of isoforms rather than a single mature sequence. Several miRNAs such as miR-17, miR-18a, miR-19b, miR-93, miR-130a, miR-30d, miR-23b, let-7a, and miR-191 generated multiple isomiRs with distinct abundance profiles [72]. While overall distributions were similar between normal and malignant cells, significant quantitative differences were observed for miR-19b, miR-23b, and miR-191, indicating that malignant transformation alters not only total miRNA expression but also the composition of individual isomiR populations, potentially influencing target gene selection and pathways controlling proliferation, invasion, and tumor progression [72].

Large-scale pan-cancer analyses further emphasize the functional importance of isomiRs in endometrial cancer. Telonis and coworkers analyzed 7466 isomiRs across TCGA and reported extensive tissue specificity, with only 48 shared among all cancer types, highlighting the highly context-dependent nature of isomiR regulation. Among overexpressed variants were members of miR-202, miR-509, miR-508, miR-514a, miR-503, and miR-483 families, whereas under expressed isomiRs included miR-200a, miR-200b, miR-200c, miR-141, miR-429, miR-205, miR-203a, miR-375, miR-21, miR-199a, miR-199b, miR-146a, miR-146b, miR-221, and miR-222, many of which are associated with epithelial cancers and EMT, suggesting that differential isomiR expression may disrupt epithelial homeostasis and facilitate invasive phenotypes [42]. Finally, data from TIE and TCGA-UCEC provide a comprehensive overview of the endometrial cancer isomiR landscape. Frequently detected variants include miR-29c-3p|0, miR-183-5p|0, miR-183-5p|+1, miR-10a-5p|0, miR-10a-5p|+1, miR-30e-5p|0, miR-30e-5p|+1, miR-21-5p|0, miR-21-3p|0, miR-182-5p|0, miR-205-5p|0, miR-199a-3p|0, miR-199b-3p|0, miR-203a-3p|0, miR-203a-3p|+1, miR-375|0, miR-200c-3p|0, along with variants from let-7, miR-125b, miR-192, miR-194, miR-142, miR-27, and miR-23 families. Many of these miRNAs regulate hormone signaling, myometrial invasion, EMT, angiogenesis, metastasis, and therapeutic response, indicating that their isomiRs substantially contribute to the biological heterogeneity of endometrial tumors [65].

## 5. Arm Switching in miRNA Regulation

The regulation of miRNAs involves not only their expression and abundance but also the selection of the active strand of the pre-miRNA, a process known as arm selection. Arm switching occurs when predominant 5p/3p strand usage changes between biological contexts [6]. Because the 5p and 3p strands have different seed sequences, changes in their predominant usage can redirect miRNA-mediated regulation toward different sets of target genes. Consequently, arm switching contributes to the regulatory plasticity of miRNAs, with direct implications for tumor progression and therapeutic resistance [39]. Arm selection is influenced by several molecular determinants, including duplex thermodynamic stability, sequence composition, and interactions with miRNA processing factors such as Dicer, TRBP, and AGO proteins.

### 5.1. Concept of 5p/3p Arm Selection

The selection of the active strand of a pre-miRNA, known as arm selection, is a critical and non-random molecular process that determines which of the two strands of an approximately 22-nucleotide miRNA duplex is incorporated into the RISC [39]. Each pre-miRNA can generate two functional strands, 5p and 3p, and the selected strand determines which mRNAs are silenced and, consequently, which gene networks are activated in a specific tissue context [6,73]. It is important to note that this process is dynamic; the chosen strand can change depending on the cell type, developmental stage, physiological state, or environmental stimuli. The strand whose 5′ region has lower thermodynamic stability is usually preferred, as this promotes initial unwinding and facilitates efficient interaction with the seed region in AGO2 within the RISC [74]. Additionally, structural elements of the fork, such as internal loops and bulges, modulate the accessibility of the ends, thereby affecting the likelihood that one strand is preferentially loaded into RISC over the other [75,76].

On the other hand, cofactor proteins such as TRBP and PACT stabilize the interaction between Dicer and the pre-miRNA, thereby increasing the accuracy of the cut and promoting the incorporation of the guide strand [20,76]. Likewise, AGO2 assesses compatibility with the seed region and ensures that the incorporated strand effectively silences the target mRNAs post-transcriptionally [75].

In many organisms, the duplex loading onto the RISC is mediated by specialized complexes. For example, in *Drosophila melanogaster*, Argonaute RISC Component 1 (AGO1) promotes miRNA loading at mismatches at positions 9–10, whereas AGO2 requires the RISC Loading Complex (RLC), formed by Dicer-2 and R2D2 [39]. Similarly, in C. elegans, members of the ALG family mediate loading. In mammals, all four Argonaute members can associate with pre-miRNAs that have internal nucleotide mismatches at positions 8–11, although there are no significant differences in loading efficiency among them [77]. However, studies show that passenger strands can be preserved and actively participate in gene regulation, generating functional silencing and contributing to regulatory heterogeneity. Consequently, the 5p/3p ratio is regulated by cellular context, tissue type, developmental stage, sex, and pathophysiological conditions. In some cases, this ratio can even be reversed, constituting an adaptive mechanism that diversifies miRNA function [39].

In conclusion, 5p/3p strand selection and arm usage are dynamic processes actively regulated by the structure of the pre-miRNA, the stability of the ends, the biogenesis machinery, and associated cofactors. Therefore, this mechanism ensures the functional plasticity of mature miRNAs, allows for diversification of their regulatory capacity, and maintains precise control over critical gene networks, with fundamental implications in differentiation, morphogenesis, and cellular adaptation [6,36,39].

### 5.2. Molecular Determinants of Arm Switching

Arm switching is a context-dependent change in the preferential selection of one miRNA arm over the other, resulting in a shift in the predominant 5p/3p strand incorporated into the RISC [36,39,78]. In contrast to the initial conception that the complementary strand was degraded immediately, recent studies have shown that these strands can persist and exert functional silencing, thereby contributing to regulatory diversification [39]. The selection of the guide strand depends on the integration of multiple molecular factors. First, the intrinsic properties of the duplex, such as the thermodynamic stability of the 5′ end and the identity of the 5′ terminal nucleotide, influence the strand loading [53,76]. The strand with the less stable 5′ end and a nucleotide compatible with AGO is preferentially incorporated, thereby facilitating unpairing of the seed region and efficient interaction with AGO2 [36,74]. In addition, the secondary structure of the pre-miRNA, in which internal loops, bulges, and stem length modulate the exposure of the ends and the Dicer cleavage site, influences the selection of the active strand and may contribute to the generation of functional isomiRs.

On the other hand, cofactor proteins such as TRBP and PACT stabilize the Dicer–pre-miRNA interaction, thereby influencing cleavage accuracy and contributing to context-dependent differences in 5p/3p strand abundance. AGO2 assesses the complementarity of the seed region to ensure that the incorporated strand effectively silences the target mRNAs [20,75,79]. Additionally, post-transcriptional modifications, such as uridylation or adenylation of the 3′ ends, can influence precursor processing and, in specific contexts, contribute to arm switching or generate functional isomiRs that expand regulatory diversity [80]. The gene duplication of miRNAs allows paralogs to evolve independently, adjusting the 5′ stability or nucleotide identity and causing changes in strand ratios, as documented in vertebrates and arthropods [81]. The Twin-Drive model integrates these factors and proposes that the MID domains of AGO act as dual sensors: one detects the stability of the 5′ end, and the other assesses nucleotide identity, thereby explaining how small structural or sequence variations induce arm switching and generate functional heterogeneity [76]. Together, these mechanisms make strand selection a flexible and adaptive process, providing a comprehensive molecular framework for understanding the functional diversification of mature miRNAs (Table 2).

### 5.3. Altered 5p/3p Arm Usage and Arm Switching in Gynecologic Cancers

Differential regulation of miRNA 5p and 3p strands should be distinguished from true arm-switching events. Dual-arm expression and dual-arm functional activity refer to the expression and biological contribution of both strands derived from the same miRNA precursor, whereas altered arm preference reflects changes in the relative abundance of the 5p and 3p strands without necessarily implying a reversal of the dominant strand. In contrast, arm switching involves a context-dependent reversal in predominant 5p/3p strand usage, in which the previously less abundant arm becomes the dominant strand. In gynecologic cancers, alterations in miRNA arm usage have been reported in ovarian, cervical, and endometrial tumors, where changes in strand selection and function may contribute to tumor-associated regulatory plasticity.

In ovarian cancer, it has been shown that the miRNA biogenesis machinery, including Dicer, Drosha, and TUT4/7, regulates the selection of the 5p and 3p strands of pre-miRNAs. The loss of TUT4/7 reduces the uridylation of pre-miRNAs and promotes the emergence of adenylated isomiRs, altering the expression of critical target genes such as *BCL2* and the let-7 family, and affecting cell proliferation and migration [22,57]. Functional studies demonstrated that modulation of Dicer processing and terminal uridylation can alter guide-strand selection and miRNA functional output, highlighting the relevance of regulated strand selection and altered miRNA arm usage in cancer [57,80].

In cervical cancer, the selection of 5p/3p miRNA strands represents an active regulatory mechanism that directly affects the expression of genes involved in proliferation, migration, invasion, and apoptosis. Various miRNA families, such as miR-7, miR-19, miR-27, and miR-34c, exhibit differential arm usage and dual-arm functional activity [82,83]. The reduced expression of the miR-7-5p strand in cervical tumors has been associated with increased expression of oncogenic targets such as focal adhesion kinase (*FAK*) and *XIAP*, affecting proliferation, migration, and invasion, whereas the miR-7-3p strand may regulate additional target networks [84,85]. In miR-27, the 3p strands retain similar regulatory functions, whereas the 5p strands exhibit diverse gene-regulatory effects [83,86]. In miR-34c, both the 5p and 3p strands exhibit tumor-suppressive activity with distinct functional effects: miR-34c-5p mainly affects proliferation, whereas miR-34c-3p shows stronger effects on migration, invasion, and apoptosis, reflecting functional diversification of both strands within the p53-associated regulatory network [87]. These findings were further supported by Córdova-Rivas and coworkers, who demonstrated that multiple 5p and 3p strands of the miR-34 family contribute to tumor-suppressive responses in cervical cancer cells [88]. The proportion of strands is modulated by the miRNA biogenesis machinery, including Dicer, Drosha, TRBP, and PACT, as well as by epigenetic factors and viral oncoproteins such as HPV16. These mechanisms contribute to context-dependent changes in miRNA arm usage and tumor heterogeneity [25,26].

In endometrial cancer, altered 5p/3p strand preference contributes to tumor progression and the reprogramming of gene networks. Additionally, epigenetic factors, interactions with lncRNAs, and the tumor microenvironment may modulate strand selection, integrating altered miRNA arm usage into complex regulatory networks that affect tumor progression and adaptation [39,89]. Importantly, arm switching and isomiR generation are interconnected processes, since changes in precursor processing and strand selection may simultaneously alter the abundance and identity of mature miRNA isoforms. Together, these findings indicate that altered 5p/3p arm usage represents an adaptive mechanism in gynecologic cancers, while confirmed arm-switching events occur in specific biological contexts with functional relevance.

## 6. Epitranscriptomic Control of miRNA Function Through Direct, Indirect, and Reciprocal Mechanisms

**Figure 2:**
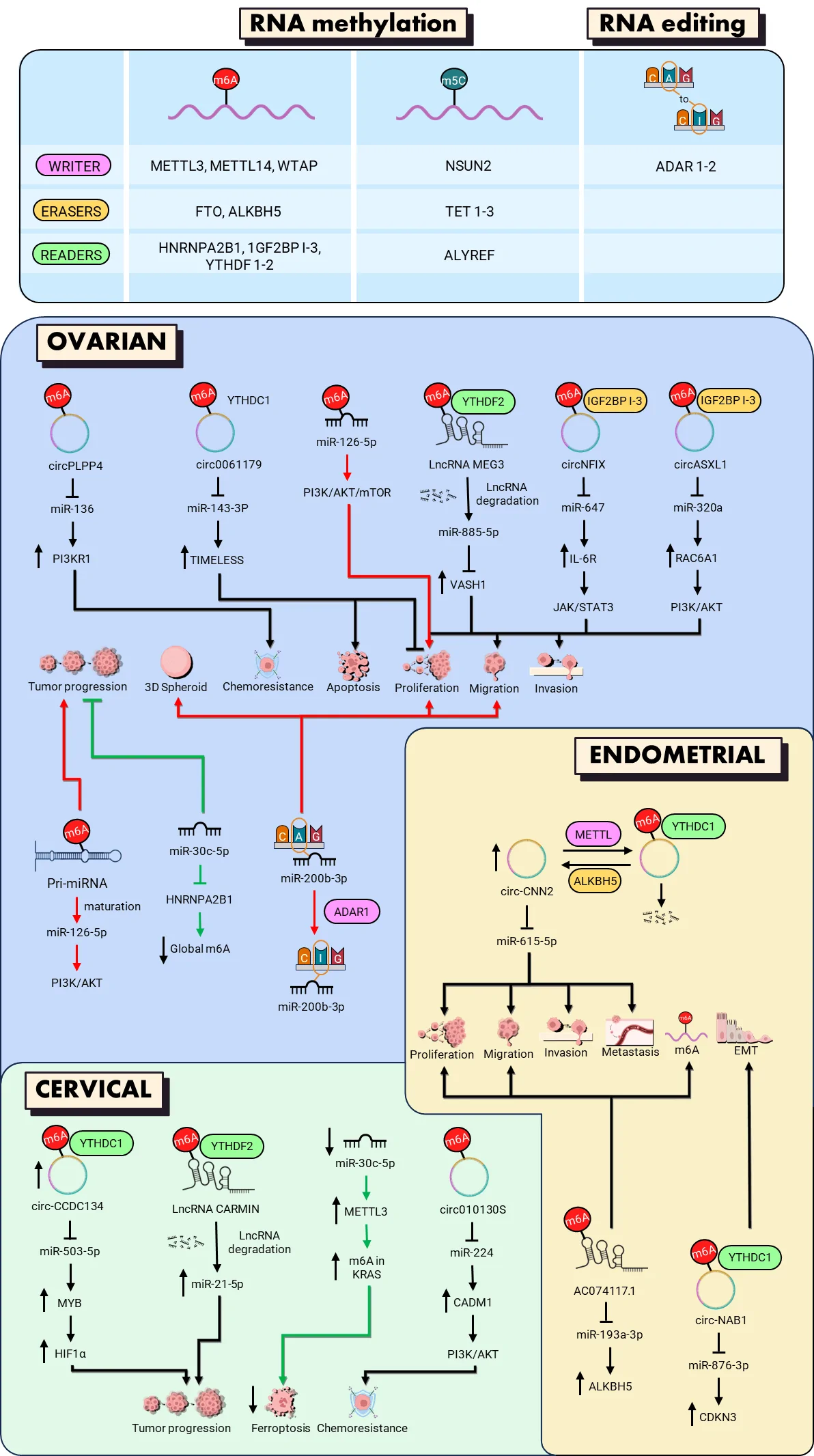
Impact of epitranscriptomic regulation on miRNA-centered co-regulatory networks in gynecologic cancers. The upper panel summarizes the main epitranscriptomic mechanisms regulating RNA modifications, including m6A, m5C, and A-to-I editing. m6A is controlled by “writers” (METTL3, METTL14, WTAP), “erasers” (FTO, ALKBH5), and “readers” (HNRNPA2B1, IGF2BP1–3, YTHDF1/2), whereas m5C and A-to-I editing are mediated by NSUN2/TET1–3/ALYREF and ADAR1/2, respectively. These mechanisms regulate RNA maturation, stability, localization, and function, influencing miRNA-mediated regulation. The central and lower panels illustrate miRNA-centered regulatory networks in ovarian, endometrial, and cervical cancers. Interactions are classified by color: black indicates indirect regulation mediated by m6A-modified circRNAs and lncRNAs that alter miRNA availability through ceRNA mechanisms; red indicates direct regulation of miRNAs or miRNA precursors; and green indicates reciprocal regulation between miRNAs and epitranscriptomic enzymes. These interconnected pathways converge on PI3K/AKT/mTOR, JAK/STAT3, KRAS, HIF1A, and ferroptosis-related signaling, affecting cell proliferation, migration, invasion, EMT, metastasis, therapeutic resistance, and apoptosis. Symbols indicate m6A RNA methylation marks, A-to-I editing events, activation (arrows), inhibition (bar-ended lines), increased or decreased expression/activity (↑/↓).

Epitranscriptomic regulation represents an additional layer of miRNA control that influences key processes such as miRNA biogenesis, stability, localization, and target interaction. This regulation can be classified into three interconnected mechanisms: (i) direct modification of miRNA precursors or mature miRNAs, affecting their processing, stability, and target recognition; (ii) indirect regulation through epitranscriptomic remodeling of miRNA-associated RNA networks, including circRNAs and lncRNAs, which modulate miRNA availability and functional activity; and (iii) reciprocal regulation, in which miRNAs influence the expression of epitranscriptomic regulators, including writers, readers, and erasers, establishing feedback mechanisms that contribute to tumor-associated RNA regulatory networks. These modifications include methylation of specific miRNA bases (m6A and m5C) and A-to-I editing, which not only affect miRNA maturation and processing but also influence their functional activity, contributing to the regulation of genes involved in tumor progression [7]. In the context of gynecologic cancers, such as ovarian, cervical, and endometrial cancers, alterations in these modifications have been associated with tumor dissemination, therapy resistance, and immune system evasion [8]. This section reviews the epitranscriptomic modifications that affect miRNAs in gynecologic cancer, highlighting their impact on the regulation of key biological processes (Figure 2).

### 6.1. Epitranscriptomic RNA Modifications Involved in miRNA Regulation

The biogenesis and function of miRNAs are regulated not only by the canonical nuclear and cytoplasmic processing machinery but also by a dynamic epitranscriptomic layer that fine-tunes miRNA maturation and processing [7]. Within this framework, chemical modifications such as m6A, m5C, and A-to-I editing act in a coordinated manner to expand miRNA diversity and increase isomiR generation, thereby enhancing regulatory plasticity [5,90]. These epitranscriptomic modifications are controlled by a regulatory system composed of writers, erasers, and readers. Writers are enzymes that catalyze the addition of chemical marks onto RNA molecules, thereby defining the modification landscape that influences miRNA biogenesis. Erasers are enzymes that catalyze the removal of these chemical marks, enabling reversible regulation of RNA function. Readers are proteins that recognize and bind these modifications, converting them into functional outcomes by modulating RNA processing, stability, or interactions with effector complexes [91,92].

In this context, m6A is deposited by the writers Methyltransferase-Like 3 (METTL3) and Methyltransferase-Like 14 (METTL14), a methyltransferase complex that catalyzes the transfer of methyl groups to adenosine residues in pri-miRNAs, thereby marking them for efficient processing. This modification enhances recognition by the Drosha–DGCR8 microprocessor complex, promoting precise cleavage and maturation. Importantly, m6A-mediated regulation can occur either directly by modifying miRNA precursors or indirectly by modifying miRNA-associated regulatory RNAs [7,93]. The reader protein heterogeneous nuclear ribonucleoprotein A2/B1 (HNRNPA2B1) binds m6A-modified RNA and facilitates its processing. In contrast, m6A removal is mediated by the erasers fat mass and obesity-associated protein (FTO) and AlkB homolog 5 (ALKBH5), two demethylases that catalyze oxidative demethylation, thereby modulating miRNA maturation rates in response to cellular context and stress signals [94,95]. Meanwhile, m5C is deposited by the writer NOP2/Sun RNA methyltransferase 2 (NSUN2), which catalyzes the addition of methyl groups to cytosine residues in pri- and pre-miRNAs. This modification stabilizes RNA secondary structure and influences the accessibility of processing sites, thereby modulating the efficiency of miRNA maturation. However, direct evidence supporting m5C-mediated regulation of miRNAs in gynecologic cancers remains limited and requires further validation. The functional outcome of m5C modification is further shaped by interactions with accessory proteins such as TRBP and PACT, which help ensure accurate Dicer processing and contribute to the fidelity of isomiR production [90,96].

On the other hand, A-to-I RNA editing, catalyzed by the RNA-editing enzymes ADAR1 and ADAR2, converts adenosine to inosine in double-stranded regions of pri- and pre-miRNAs, thereby altering the secondary structure and complementarity of the seed region. As a result, A-to-I editing can alter strand selection, generate functional isomiRs, and modify target specificity, enabling miRNAs to expand their repertoire of gene regulation and respond to dynamic cellular signals [97,98].

### 6.2. Impact on miRNA Maturation, Stability, RISC Loading, and Target Recognition

Epitranscriptomic modifications not only influence miRNA maturation but also determine the functional capacity of mature miRNAs to regulate their target genes and participate in complex signaling networks. For example, m6A can affect the affinity of miRNAs for AGO2, reducing intracellular miRNA activity and promoting their export to extracellular vesicles for intercellular communication, a mechanism that broadens the scope of gene regulation and cellular plasticity [99]. These modifications can also influence guide strand selection and the 5p/3p ratio, allowing a single precursor to generate functionally distinct variants depending on the cellular context. The presence of isomiRs confers on miRNAs the ability to regulate a broader spectrum of genes, diversifying the cellular response to internal and external stimuli [8,97].

In addition, modification readers such as YTHDF1–3 and YTHDC1 recognize modified RNAs and influence their stability, localization, and processing, thereby potentially affecting miRNA availability, RISC incorporation, and target recognition. These mechanisms can influence silencing efficiency and modulate critical pathways involved in cell proliferation, apoptosis, and differentiation [100]. This level of regulation allows cells to dynamically adjust their post-transcriptional regulatory landscape, contributing to adaptive processes and, in a tumor context, to progression, drug resistance, and immune evasion [7]. Finally, the combination of RNA modifications and reader proteins contributes to the generation of functional miRNA diversity and context-dependent regulation [101]. This refined control of mature miRNA function constitutes an essential mechanism for coordinating complex cellular responses and explains the heterogeneity observed in tumor cells and physiological tissues [102].

### 6.3. Evidence in Gynecologic Cancers

Experimental evidence indicates that epitranscriptomic mechanisms contribute to the regulation of miRNA-mediated networks in gynecologic cancers. Although direct chemical modifications of miRNAs themselves remain relatively underexplored in these malignancies, available studies indicate that epitranscriptomic regulation may influence miRNA-centered networks through three interconnected mechanisms: direct modification of miRNA molecules or their precursors, indirect regulation through m6A-modified miRNA-associated non-coding RNAs, including circRNAs and lncRNAs, and reciprocal regulation in which miRNAs modulate the expression of epitranscriptomic regulators such as writers, readers, and erasers. Importantly, these mechanisms should not be considered equivalent in terms of experimental evidence. While direct modifications of miRNAs or their precursors provide evidence for molecular regulation at the miRNA level, a substantial proportion of m6A-related studies in gynecologic cancers involve modification of miRNA-associated circRNAs and lncRNAs, representing indirect regulation of miRNA availability and activity (Table 3).

In ovarian cancer, epitranscriptomic regulation of RNA molecules, particularly through m6A modification, influences miRNA activity and the function of non-coding RNA networks involved in proliferation, migration, invasion, drug resistance, DNA repair, and immune evasion. Recent studies have shown that the circRNA circNFIX, whose stability is reinforced by m6A and which acts as a sponge for miR-647, increases IL-6R expression and activates the Janus kinase 1/signal transducer and activator of transcription 3 (JAK1/STAT3) pathway, thereby increasing PD-L1 expression and promoting proliferation, metastasis, and immune evasion in cell models and xenografts. The regulation of circNFIX depends on the recognition of m6A by IGF2BP1–3 proteins, and co-silencing of miR-647 reverses these oncogenic effects, demonstrating how epitranscriptomic remodeling of circRNAs can modulate miRNA availability and downstream signaling pathways [103]. Additionally, circASXL1, stabilized by METTL3/IGF2BP1 through m6A, sponges miR-320d, thereby increasing *RACGAP1* expression and activating the PI3K/AKT pathway. This promotes cell proliferation, migration, and invasion and is associated with poor prognosis, highlighting the role of m6A-modified non-coding RNAs in indirectly regulating miRNA-mediated gene expression [93]. In the context of DNA repair and apoptosis, hsa_circ_0061179 acts as an m6A-modified sponge for miR-143-3p, which directly regulates *TIMELESS* expression. The m6A modification mediated by METTL3 and recognized by YTHDC1 promotes the stabilization and cytoplasmic localization of hsa_circ_0061179, enhancing its ability to sequester miR-143-3p. Consequently, upregulation of hsa_circ_0061179 reduces miR-143-3p availability, leading to increased *TIMELESS* expression, whereas hsa_circ_0061179 knockdown or miR-143-3p overexpression suppresses ovarian cancer cell proliferation, increases DNA damage, and promotes apoptosis [104]. Chemotherapy resistance is also modulated by m6A-modified RNAs. circPLPP4, stabilized by m6A, sequesters miR-136, increasing the expression of PIK3R1 and promoting cisplatin resistance [105]. Interestingly, pseudogene-derived lncRNAs also participate in this network. For example, RPS15AP12, whose expression is increased by FTO-mediated demethylation, acts as a ceRNA for RPS15A by sequestering miR-96-3p, thereby enhancing proliferation and metastasis and suppressing the innate immune response [106]. Another example is the tumor-suppressor lncRNA MEG3, which is methylated by METTL3 and subsequently degraded by YTHDF2. MEG3 functions as a sponge for miR-885-5p, thereby relieving its inhibitory effect on VASH1 and ultimately suppressing proliferation, migration, and invasion in ovarian cancer cells [107].

Beyond indirect regulation through modified non-coding RNAs, epitranscriptomic mechanisms can also directly affect miRNA biogenesis and function. In addition, the writer protein WTAP is regulated by HIF-1α under hypoxia, promoting the maturation of miRNAs, such as the miR-200 family, which in turn affect glycolysis through HK2, integrating metabolic adaptation and tumor progression [108]. Finally, miRNAs can directly regulate the m6A machinery. miR-30c-5p inhibits the reader HNRNPA2B1, decreasing the overall level of m6A and limiting proliferation, migration, and invasion [109]. Additionally, the maturation of miR-126-5p is promoted by METTL3 through m6A modification of pri-miR-126-5p, thereby activating the phosphoinositide 3-kinase/protein kinase B/mechanistic target of rapamycin (PI3K/Akt/mTOR) pathway and promoting tumor progression [110]. Importantly, epitranscriptomic regulation in ovarian cancer is not limited to m6A. A-to-I editing of miR-200b-3p represents one of the best-characterized examples of direct miRNA modification. Increased A-to-I editing correlates with poorer overall survival and promotes cell proliferation, migration, and 3D spheroid formation, whereas ADAR1 knockdown attenuates these effects, demonstrating that RNA editing can directly reshape miRNA function and target recognition [64]. Together, these findings demonstrate that epitranscriptomic regulation influences miRNA-centered networks in ovarian cancer through complementary mechanisms, including direct modulation of miRNAs and indirect remodeling of miRNA-associated RNA interactions. These regulatory layers control proliferation, metastasis, drug resistance, DNA repair, metabolic adaptation, and immune evasion in ovarian cancer, presenting multiple opportunities for targeted therapeutic intervention.

In cervical cancer, recent studies have demonstrated that epitranscriptomic regulation of miRNA-centered networks coordinates multiple aspects of tumor biology. Specifically, the lncRNA CARMN has been identified as a tumor suppressor whose expression is reduced by m6A-mediated degradation dependent on the reader YTHDF2. Simultaneously, miR-21-5p inhibits CARMN through direct binding, thereby promoting tumor progression. This process illustrates how m6A-modified non-coding RNAs and miRNAs interact to regulate the stability and availability of regulatory molecules, highlighting these mechanisms as potential therapeutic targets [111]. Likewise, the miR-30c-5p/METTL3/*KRAS* axis demonstrates that miRNAs can regulate components of the epitranscriptomic machinery rather than being direct targets of RNA modification. Gong and coworkers demonstrated that reduced miR-30c-5p expression in cervical cancer cells promotes the overexpression of METTL3, an m6A writer that modifies KRAS, a regulator of ferroptosis. The inhibition of the METTL3/KRAS axis by miR-30c-5p promotes ferroptosis and reduces proliferation and metastasis in xenograft models [112]. Complementarily, epitranscriptomically modified circRNAs represent an additional mechanism through which miRNA activity is indirectly regulated. For example, hsa_circ_0101308, whose stability depends on m6A, sponges miR-224; a decrease in its expression releases this miRNA to repress *CADM1* and activate the PI3K/AKT pathway, which contributes to chemoresistance [113]. Furthermore, the circRNAs circCCDC134 and the lncRNA HEIH demonstrate how m6A-modified regulatory RNAs integrate nuclear and cytoplasmic signals, affecting miRNA availability and downstream oncogenic pathways, including the activation of hypoxia-inducible factor 1-alpha (HIF1-α) and epidermal growth factor receptor (EGFR), thereby contributing to metastasis and tumor stem cell-like properties [114,115]. Together, these findings support an integrative model in which epitranscriptomic modifications regulate the stability, localization, and function of lncRNAs and circRNAs, thereby influencing miRNA-mediated regulation in cervical cancer. Thus, tumor progression, chemoresistance, metastasis, and the acquisition of stem cell-like features are understood as phenomena mediated by dynamic epitranscriptomic networks that involve both direct regulation of RNA molecules and indirect modulation of miRNA activity.

In endometrial cancer, epitranscriptomic regulation of miRNA-centered networks, particularly through m6A modification, plays an important role in controlling the expression and function of non-coding RNAs, modulating proliferation, migration, invasion, epithelial–mesenchymal transition (EMT), and cell cycle progression. m6A regulators, such as insulin-like growth factor 2 mRNA-binding proteins (IGF2BP1) and METTL16, are differentially expressed in endometrial carcinoma and uterine carcinosarcoma, correlating with tumor grade, age, and survival, and enabling the construction of prognostic risk signatures that reflect the clinical relevance of epitranscriptomic regulation in tumor progression [116]. Interestingly, the m6A-regulated circRNA, circ-NAB1, is overexpressed in endometrial cancer tissues and cells. The demethylase ALKBH5 regulates its expression through a YTHDF2-dependent mechanism. Circ-NAB1 promotes proliferation, migration, invasion, EMT, and cell cycle progression by acting as a sponge for miR-876-3p, thereby derepressing its downstream target, *CDKN3*. Importantly, CDKN3 overexpression rescues the inhibitory effects observed following circ-NAB1 knockdown, confirming the functional relevance of the m6A-regulated circRNA/miRNA regulatory axis in endometrial cancer progression [117]. Similarly, circCNN2, an m6A-regulated circular RNA, is stabilized by ALKBH5 and degraded through YTHDF2-mediated recognition. CircCNN2 sponges miR-615-5p, preventing the repression of MYH14 and promoting cell proliferation, migration, invasion, and survival; its silencing induces apoptosis and reduces tumor growth and metastasis in vivo [118]. These findings highlight how epitranscriptomic remodeling of circRNAs can indirectly influence miRNA availability and downstream gene regulation. LncRNAs are also part of these m6A-regulated networks. For example, AC074117.1, through its interaction with miR-193a-3p, attenuates ALKBH5 activity, promoting cell proliferation and migration. This demonstrates that not only circRNAs but also lncRNAs act as epitranscriptomic-regulated competing endogenous RNA (ceRNA) networks that modulate miRNA-mediated pathways in tumor progression [119]. Together, these studies show that epitranscriptomic regulation in endometrial cancer involves a hierarchical network in which m6A modifies the stability and fate of circRNAs and lncRNAs, which subsequently regulate miRNA availability and activity, ultimately affecting effector genes involved in cell cycle control, EMT, cell proliferation, and invasion.

**Table 3:** Direct, indirect, and reciprocal epitranscriptomic regulation of miRNA function in gynecologic cancers.

Cancer Type	Regulatory Category	Regulation Axis	miRNA Involved	Mechanism	Reference
Ovarian cancer	Indirect regulation	circNFIX/ miR-647/ IL-6R	miR-647	m6A-mediated stabilization of circNFIX enhances miR-647 sponging.	[103]
	Indirect regulation	circASXL1/miR-320d/ RACGAP1	miR-320d	m6A-mediated stabilization of circASXL1 enhances miR-320d sponging activity.	[93]
	Indirect regulation	circ_0061179/miR-143-3p/TIMELESS	miR-143-3p	m6A modification promotes circRNA stabilization and cytoplasmic localization, reducing miR-143-3p availability.	[104]
	Indirect regulation	circPLPP4/ miR-136/ PIK3R1	miR-136	m6A modification enhances circPLPP4 stability and miRNA sponging.	[105]
	Indirect regulation	lncRNA RPS15AP12/miR-96-3p	miR-96-3p	FTO-mediated demethylation increases RPS15AP12 expression and miR-96-3p sponging activity.	[106]
	Indirect regulation	lncRNA MEG3/miR-885-5p/VASH1	miR-885-5p	METTL3-mediated m6A modification promotes MEG3 degradation, modulating miR-885-5p availability.	[107]
	Direct regulation	WTAP/HIF-1α pathway	miR-200 family	Hypoxia-induced WTAP promotes miRNA maturation through epitranscriptomic regulation by enhancing miRNA processing.	[108]
	Reciprocal regulation	miR-30c-5p/HNRNPA2B1	miR-30c-5p	miR-30c-5p regulates the m6A reader HNRNPA2B1, affecting m6A-dependent RNA regulation.	[109]
	Direct regulation	ADAR-mediated RNA editing	miR-200b-3p	ADAR-mediated A-to-I editing alters miR-200b-3p sequence and target recognition, generating a distinct miRNA variant.	[64]
	Direct regulation	METTL3-mediated m6A modification	pri-miR-126-5p	METTL3-mediated m6A modification enhances pri-miR-126-5p processing and mature miRNA production.	[110]
Cervical cancer	Indirect regulation	lncRNA CARMN/miR-21-5p	miR-21-5p	m6A-dependent regulation alters CARMN stability and modulates the availability of miR-21-5p.	[111]
	Reciprocal regulation	miR-30c-5p/METTL3	miR-30c-5p	miR-30c-5p suppresses METTL3 expression, modulating m6A-dependent regulation of KRAS.	[112]
	Indirect regulation	circ_0101308/miR-224/CADM1	miR-224	m6A modification regulates circ_0101308 stability and miR-224 sponging activity.	[113]
	Indirect regulation	circCCDC134/miR-503-5p/MYB	miR-503-5p	m6A-mediated regulation of circCCDC134 affects miR-503-5p availability.	[114]
Endometrial cancer	Indirect regulation	circ-NAB1/miR-876-3p/CDKN3	miR-876-3p	m6A-dependent regulation alters circ-NAB1 stability and modulates miR-876-3p availability.	[117]
	Indirect regulation	circCNN2/miR-615-5p	miR-615-5p	m6A-mediated regulation modifies circCNN2 stability and miR-615-5p sponging activity.	[118]
	Indirect regulation	lncRNA AC074117.1/miR-193a-3p	miR-193a-3p	m6A-mediated regulation of lncRNA AC074117.1 modulates miR-193a-3p availability via ceRNA activity.	[119]

## 7. Clinical Relevance and Translational Perspectives of Non-Canonical miRNA Regulation

The identification of isomiRs, altered 5p/3p strand utilization, arm-switching events, and RNA epitranscriptomic modifications has expanded the clinical interpretation of miRNA biology beyond canonical expression profiles. These regulatory layers generate additional molecular diversity that can influence tumor heterogeneity and disease progression. High-throughput sequencing studies have demonstrated that mature miRNA populations frequently contain multiple non-canonical variants, emphasizing the importance of considering the complete miRNA isoform landscape in cancer biology [42,68]. However, despite their potential clinical relevance, current evidence is based on sequencing analyses, retrospective cohort studies, and experimental models, and further validation is required before routine clinical implementation.

### 7.1. Candidate Biomarkers for Tumor Classification and Molecular Stratification

IsomiR profiles represent an additional layer of molecular information that may complement conventional miRNA-based analyses. In ovarian cancer, specific isomiR signatures have been associated with distinct histological subtypes, including differences between high-grade serous ovarian cancer (HGSOC) and low-grade serous ovarian cancer (LGSOC), suggesting their potential contribution to molecular tumor classification [63]. Similarly, integrative analyses of TCGA datasets and the TIE identified tumor-associated isomiR patterns in cervical and endometrial cancers, supporting their potential as molecular signatures specific to these tumor contexts [68].

Additional non-canonical features may further contribute to molecular characterization. A-to-I RNA editing of miR-200b-3p mediated by ADAR has been associated with HGSOC and tumor-related features linked to disease progression, suggesting that edited miRNAs may provide complementary information to canonical miRNA profiles [64,120]. Differential activity of miR-34c-3p and miR-34c-5p in cervical cancer demonstrates that both mature strands can exert independent biological effects, highlighting the functional relevance of strand-specific miRNA regulation [87,88]. However, these findings remain exploratory and require independent validation before clinical application.

### 7.2. Therapeutic Targeting of miRNA Variants

The identification of isomiRs possessing unique seed sequences has opened new opportunities for highly specific therapeutic interventions in gynecologic cancers. For instance, miR-9-alt, a 5′-isomiR generated by alternative Drosha cleavage, has a seed sequence distinct from canonical miR-9, enabling it to regulate over 500 unique target genes and representing a potential therapeutic target independent of the canonical miRNA [66].

Arm switching also represents an emerging therapeutic avenue, as manipulating strand selection can redirect miRNA activity toward regulatory networks that suppress tumor progression. Experimental studies indicate that modulating Dicer processing, Argonaute loading, or terminal uridylation can alter arm selection and influence tumor cell proliferation, migration, and invasion [57,70]. Although still largely experimental, therapeutic control of arm-switching events may offer a novel mechanism for reprogramming oncogenic miRNA networks in cervical, ovarian, and endometrial cancers.

RNA modification machinery constitutes an additional layer of actionable therapeutic targets. Inhibiting ADAR-mediated A-to-I editing can suppress the production of oncogenic edited miRNAs while preserving the function of tumor-suppressive canonical variants, providing a strategy to selectively modulate pathogenic miRNA activity [88]. Similarly, altering TUT4/TUT7-mediated uridylation impacts isomiR biogenesis. In ovarian cancer cells, loss of TUT4/7 activity reshapes the isomiR landscape, reducing proliferation and migration, and suggesting that these enzymes represent actionable points for therapeutic intervention [57].

Collectively, these strategies illustrate how targeting isomiRs, arm switching, and RNA modifications offer a multi-layered approach to fine-tune miRNA function in gynecologic cancers. By exploiting the molecular diversity generated through post-transcriptional modifications, it becomes possible to design interventions that selectively restore tumor-suppressive networks or suppress oncogenic pathways, paving the way for highly precise RNA-based therapeutics.

### 7.3. Limitations for Clinical Translation

Despite the growing evidence supporting the clinical utility of isomiRs, arm-switching events, and RNA-modified miRNAs as biomarkers and therapeutic targets, several important challenges continue to limit their translation into routine clinical practice. One of the primary obstacles to clinical translation is the lack of standardized methodologies for isomiR detection, quantification, annotation, and clinical interpretation. Differences among sequencing platforms, analytical pipelines, and reporting criteria limit reproducibility and hinder the establishment of clinically applicable signatures [66]. Furthermore, the complexity and diversity of miRNA isoforms make clinical interpretation challenging. A single miRNA precursor can generate multiple isomiRs through alternative processing events, terminal modifications, and RNA editing, while changes in strand selection can further expand functional diversity, making it challenging to determine which variants represent clinically relevant biomarkers. As a result, distinguishing biologically relevant variants from sequencing artifacts or low-abundance byproducts remains a significant challenge, particularly when many isoforms differ by only one or a few nucleotides.

Another major limitation is the relatively limited functional characterization of the vast majority of identified isomiRs. Although large-scale sequencing studies have cataloged thousands of isomiRs across different cancer types, only a small subset has been experimentally validated, and the biological significance of most arm-switching events and RNA editing-derived miRNA variants remains poorly understood. In addition, robust clinical validation studies are still lacking for many proposed isomiR biomarkers, underscoring the need for large, multicenter prospective studies to establish their clinical utility, reproducibility, and cost-effectiveness. Finally, the development of isomiR-based therapeutics faces important regulatory and technical hurdles, including the optimization of delivery systems, minimization of off-target effects, long-term safety assessment, and standardization of liquid biopsy methodologies. Addressing these challenges will be essential before isomiR-, arm-switching-, and RNA modification-based approaches can be fully integrated into precision oncology and routine clinical practice [121,122].

## 8. Challenges and Future Directions

Despite significant advances in our understanding of miRNA biology and epitranscriptomic regulation, important biological and methodological questions remain unresolved. Although key factors underlying arm switching and epitranscriptomic changes have been identified, the molecular mechanisms governing strand selection, precursor processing, and context-dependent regulatory effects remain incompletely understood. The interactions between precursor structure, cofactor proteins, and strand competition require further study, particularly in human tumor tissues, where regulatory plasticity may differ from that observed in in vitro models [39,123].

Another major challenge is understanding how tumor-specific environments influence isomiR generation, arm selection, and RNA modification patterns. The dynamics of these regulatory processes may vary according to cellular context, tumor heterogeneity, and external stimuli, highlighting the need for functional studies that integrate molecular mechanisms with tumor biology [7].

In addition, the development of robust bioinformatics approaches that integrate isoform-resolved sequencing with epitranscriptomic modification profiles remains essential. Such tools will enable more precise characterization of miRNA functional diversity, identification of biologically relevant variants, and elucidation of regulatory networks associated with specific miRNA strands or chemical modifications [8].

## 9. Conclusions

Accumulating evidence indicates that miRNA function in gynecologic cancers extends beyond the canonical miRNA sequences. IsomiR generation, miRNA strand selection, arm switching, and epitranscriptomic regulation represent additional regulatory layers that contribute to miRNA functional diversity and tumor heterogeneity. Among these regulatory layers, isomiR profiles have been extensively described through high-throughput sequencing studies, including large-scale cancer datasets and specialized resources, revealing tumor-associated patterns and potential functional implications. However, the biological relevance of many individual isoforms requires further experimental validation. Similarly, miRNA strand selection and arm switching represent important mechanisms of functional diversification, although changes in 5p/3p abundance should be distinguished from true arm-switching events, which require evidence of context-dependent changes in predominant strand usage. Epitranscriptomic regulation involves multiple mechanisms, ranging from direct modification of miRNAs or their precursors to indirect modulation of miRNA activity through modified circRNAs and lncRNAs. Although these regulatory layers offer promising opportunities for biomarker discovery and therapeutic development, their clinical translation remains limited by methodological variability, insufficient functional characterization, and the lack of prospective clinical validation. Future studies integrating isoform-resolved sequencing, epitranscriptomic profiling, and functional approaches will be required to determine which miRNA variants and regulatory mechanisms have the greatest clinical relevance. Overall, these findings support the concept that miRNA function in gynecologic cancers is dynamic and context-dependent, highlighting the need to move beyond canonical miRNA expression toward a more comprehensive understanding of miRNA regulatory networks.

## Figures and Tables

**Table 1:** Types of isomiRs in gene regulation.

Type of isomiR	Molecular Origin	Main Feature	Functional Consequence
5′ isomiRs	Alternative Drosha or Dicer cleavage at the 5′ end	Shift in seed region sequence	Reprograms the specificity of interaction with mRNAs and generates new repertoires of target genes
3′ isomiRs	Alternative cleavage or exonucleolytic trimming at the 3′ end	Length variation without seed alteration	Modulates stability, AGO loading, and RNA degradation
Non-templated isomiRs	Post-transcriptional nucleotide addition by TUT4/7 and related transferases	3′ addition of non-genomic nucleotides (A/U)	Regulates stability, maturation, and decay of miRNAs
Polymorphic isomiRs	RNA editing (ADAR) or genetic variation (SNPs)	Internal nucleotide substitutions	Alters binding affinity and target recognition

**Table 2:** Molecular determinants of miRNA arm switching.

Determinant	Mechanism	Functional Outcome
Thermodynamic asymmetry of miRNA duplex	Differential stability at the 5′ ends of miRNA duplex determines strand selection during RISC loading	Defines the dominant (guide) strand and suppresses the passenger strand
Drosha/Dicer processing precision	Variability in cleavage site selection generates duplexes with shifted ends	Alters seed identity and changes which strand is preferentially incorporated into RISC
RNA-binding proteins (RBPs)	Proteins such as TRBP and PACT interact with the Dicer complex and influence strand sorting	Biased selection toward either the 5p or 3p strand in a context-dependent manner
3′ tailing (TUT4/7-mediated uridylation)	Terminal modifications destabilize one strand or alter duplex asymmetry	May alter strand preference and, in specific contexts, contribute to arm switching, thereby changing functional miRNA output.
RNA editing (ADAR-mediated A-to-I conversion)	Structural changes in pri-/pre-miRNA alter duplex stability and base pairing	Shifts strand preference and generates alternative functional miRNA species

## Data Availability

No new data were created or analyzed in this study. Data sharing is not applicable to this article.

## Abbreviations

The following abbreviations are used in this manuscript:

ADAR	Adenosine deaminase acting on RNA
AGO1	Argonaute RISC Component 1
AGO2	Argonaute RISC Catalytic Component 2
AGO3	Argonaute RISC Catalytic Component 3
AKT	Protein kinase B
ALKBH5	Alkb homolog 5
A-to-I	Adenosine-to-Inosine
BCL2L11	Bcl-2-like protein 11
BMI	Body Mass Index
CASP3	Caspase 3
CDS	Coding sequence
DGCR8	DiGeorge syndrome critical region 8
DHX9	DExH-Box Helicase 9
EGFR	Epidermal growth factor receptor
EMT	Epithelial-to-mesenchymal transition
EOC	Epithelial ovarian cancer
FAK	Focal Adhesion Kinase
FTO	Fat mass and obesity associated protein
GLD2	Germline development 2
HGSOC	High-grade serous ovarian carcinoma
HIF1A	Hypoxia-inducible factor 1-alpha
HNRNPA2B1	Heterogeneous nuclear ribonucleoprotein A2/B1
HPV	Human papillomavirus
HPV16	Human Papillomavirus type 16
IGF2BP1	Insulin-like growth factor 2 mRNA-binding proteins
JAK	Janus kinase 1
LGSOC	Low-grade serous ovarian carcinoma
m5C	5-methylcytosine
m6A	N6-methyladenosine
MAP2	Microtubule-associated protein 2
METTL14	Methyltransferase-Like 14
METTL3	Methyltransferase-Like 3
MicroRNAs	miRNAs
MMP2	Matrix metalloproteinase-2
MMP9	Matrix metalloproteinase-9
mRNAs	messenger RNAs
mTOR	Mechanistic target of rapamycin
MTPAP	mitochondrial poly(A) polymerase
NSUN2	NOP2/Sun RNA methyltransferase 2
NTA(s)	non-templated nucleotide addition(s)
PACT	Protein activator of PKR
PAPD4	poly(A) RNA polymerase D4
PAPD5	poly(A) RNA polymerase D5
PARN	poly(A)-specific ribonuclease
PI3K	phosphoinositide 3-kinase
Pre-miRNA	precursor miRNA
Pri-miRNAs	primary miRNAs
RISC	RNA-induced silencing complex
RLC	RISC Loading Complex
SNP(s)	Single nucleotide polymorphism(s)
STAT3	Signal transducer and activator of transcription 3
TDP-43	TAR DNA-binding protein 43
TIE	Tumor IsomiR Encyclopedia
TRBP	TAR RNA-binding protein
TUT1	Terminal uridylyl transferase 1
TUT4	Terminal uridylyl transferase 4
TUT7	Terminal uridylyl transferase 7
XIAP	X-linked inhibitor of apoptosis
YTHDC1	YTH domain-containing protein 1
YTHDF	YTH N6-methyladenosine RNA-binding protein
